# Measles outbreak in an office building in the crowded Metropolis of Beijing, China

**DOI:** 10.1186/s12879-019-4404-6

**Published:** 2019-09-03

**Authors:** Zhen Li, Zheng Zhang, Fang Wang, Rui Wei, Jianhong Zhao, Fang Liu

**Affiliations:** Chaoyang District Center for Disease Control and Prevention, Beijing, China

**Keywords:** Measles outbreak, Outbreak response immunization, Active surveillance, Beijing

## Abstract

**Background:**

Although worldwide measles elimination achieved great progress for decades, outbreaks were still reported in certain countries. This study describes the epidemiologic features of a substantial measles outbreak in an office building in Beijing and explores control strategies in a crowded city.

**Methods:**

We performed descriptive analyses of data on demographic characteristic, laboratory testing and epidemiological information.

**Results:**

From February 25 to March 28, 2016, 43 outbreak-related measles cases occurred in an office building in Beijing. The total crude attack rate was 1.20% in the building. The age range of patients was 23 to 45 years old, of whom 30 (69.8%) were migrants and 5 (11.6%) were vaccinated but without documentation. The attack rate of the department and the company of the source case was 22.73 and 11.86%, respectively. The attack rate in the building was 1.78%, except for the commercial center on the lower floors, which was 0.34%. Of the 43 measles cases, only 19 cases (53.5%) were reported by hospitals through the National Notifiable Disease Reporting System (NNDRS), and the rest were found through active surveillance. Outbreak response immunization was conducted for 6216 persons.

**Conclusions:**

Office buildings in crowded metropolis are prone to large-scale measles outbreaks, and require a rapid outbreak response. Early Outbreak response immunization and active surveillance are important strategies to control outbreaks such as the one reported herein.

## Background

In 2006, China endorsed the 2006–2012 national action plan for measles elimination following global goals proposed by the World Health Organization (WHO) [1, 2]. China primarily aimed to increased vaccination coverage (to > 95%), combined with strengthening of surveillance systems and infection control. During this period, measles incidence decreased from 99.5 per million persons in 2008 to 4.6 per million in 2012 [1]. In Beijing, supplementary immunization activities (SIAs) have been conducted among migrant preschoolers following annual spring festival period since 2004, and these activities have effectively improved the vaccination coverage among these children [3]. Additionally, annual spring measles vaccination of migrant workers has been conducted in Beijing, with the coverage reaching more than three million persons since December 2003. The percentage of migrant cases had decreased significantly from 72.6% in 2004 to 49.4% in 2016. In Beijing, the measles incidence has reduced from 210.6 per million in 2005 to 58.1 per million in 2016 [4, 5], but further and extensive efforts are needed to achieve global measles elimination.

According to the Beijing Statistical Yearbook 2016, the permanent resident population in the metropolis had reached 21.7 million in 2015, with a migrant population of 8.23 million. Population density was 1323 per square mile, ranking the third in China after Shenzhen and Shanghai. The large population, high density and human mobility collectively constitute unfavorable factors for measles control in Beijing [6–8]. Recent data show that the proportion of measles cases among individuals of 20 to 44 years old in Beijing increased from 37.2% in 2005 to 73.5% in 2016. Investigation on antibody levels in serum among a healthy population in Beijing in 2012 showed that the positive rate of measles immunoglobulin G (IgG) was 86.7% among this age group [9]; lower than the herd immunity threshold of 95% needed for elimination [10, 11].A trend in outbreaks in Beijing in recent years is that white-collar workers account for the majority of patients rather than blue-collar. Outbreak settings are also shifting from locations such as factories and large-scale markets to office buildings [3, 12, 13]. The present study describes the epidemiologic features of a measles outbreak in an office building, and explores prevention and control strategies for measles among adults in a crowded city.

## Methods

### Case and outbreak definitions

Herein, a measles case was defined as a person who either had a laboratory-confirmed measles infection with positive serology for measles immunoglobulin M (IgM), and/or presence of measles RNA, or had an acute febrile rash illness and was epidemiologically linked to a person with a laboratory-confirmed case. This is consistent with the WHO clinical case definition. In accordance with China’s national measles surveillance guidelines [14], a measles outbreak was defined as the occurrence of two or more measles cases in a group setting (e.g., community, school, company and building) within a 10-day time frame. There being no epidemiologically linked case within 21 days (maximum incubation period) from the onset of the last-reported case represented the end of the outbreak.

### Laboratory testing

Serum specimens and throat swabs were obtained and tested by Beijing’s measles laboratory network for confirmation. Serology for immunoglobulin M (IgM) used a commercial enzyme linked immunosorbent assay (ELISA, Virion/Serion GmbH, Würzburg, Germany). If the IgM result from serum collected 0–3 days after rash onset was negative or suspect, another serum specimen was collected at 4–28 days. The throat swab was obtained within 5 days from rash onset for viral testing using real-time reverse transcription polymerase chain reaction (RT-PCR) (Shuoshi Company, Jiangsu). The measles virus was isolated from clinical specimens using the Vero/hSLAM cell line. According to the national measles surveillance guidelines of China, the isolates were send to the China CDC and provincial CDC for further genotyping identification. If isolates were not obtained, then N450 sequences were obtained directly from for further genotyping. The methods on virus isolation and genomic sequencing analyses were described in these papers [15–18].

### Data collection

Measles cases were diagnosed and reported by a hospital through the National Notifiable Disease Reporting System (NNDRS). During the outbreak, all cases identified via active surveillance were reported by the local Center for Disease Control (CDC). Face-to-face interviews and standardized case investigation forms were used to collect information on demographic characteristics, clinical presentation, epidemiological contact and vaccination.

### Statistical analyses

Descriptive analyses were performed with frequencies and proportions, using R 3.1 statistical software.

## Results

### Outbreak setting

The measles outbreak occurred in an office building, located in a Beijing business district near a main road at the intersection of two subway lines. The office building has 28 floors, of which the sixth floor and below are part of commercial center. There is no passage between the office building and the commercial center. The elevators directly service the seventh floor and above. There are more than 50 companies and 2136 people on the 7–28 floors of the office building and 1460 staff in the commercial center. Company A, where the source case was found, occupies the entire 11th floor, and has more than 20 departments with 236 people, of which department A has 22 people. The working area of department A is open, using a 1.5-m-high partition, divided into many personal working areas of about 2.5 square meters each. The office building and commercial center each has separate dedicated entrances. The entrance to the commercial center is on the east side of the building, facing the traffic flow. The entrance of the office building is on the west side.

### Chronology of the outbreak

The index case was a 36-year-old woman in department A of company A. She experienced fever on March 5, and was reported by a hospital in the NNDRS when rash developed on March 8. Moreover, the hospital reported to the local CDC both by telephone and the Infectious Symptom Surveillance System in Beijing because several colleagues of the index case showed similar symptoms. The local CDC responded quickly after receiving the report, with actions including investigation, collecting samples and vaccination.

The source patient, a 41-year-old migrant man, worked in the same department as the indicated case. He had traveled to his hometown during the Chinese Spring Festival (Feb 9, 2016 to Feb 16, 2016). His hometown was in another province, which has high incidence of measles. He began having fever and rash on February 25, and received intravenous fluids for drug rash and pneumonia at a local hospital on February 27. He was not reported as an infectious disease and worked in the company on February 23–26 and 29. From March 3, there were several successive cases of fever and rash within his company and mainly concentrated in department A.

**Fig. 1:**
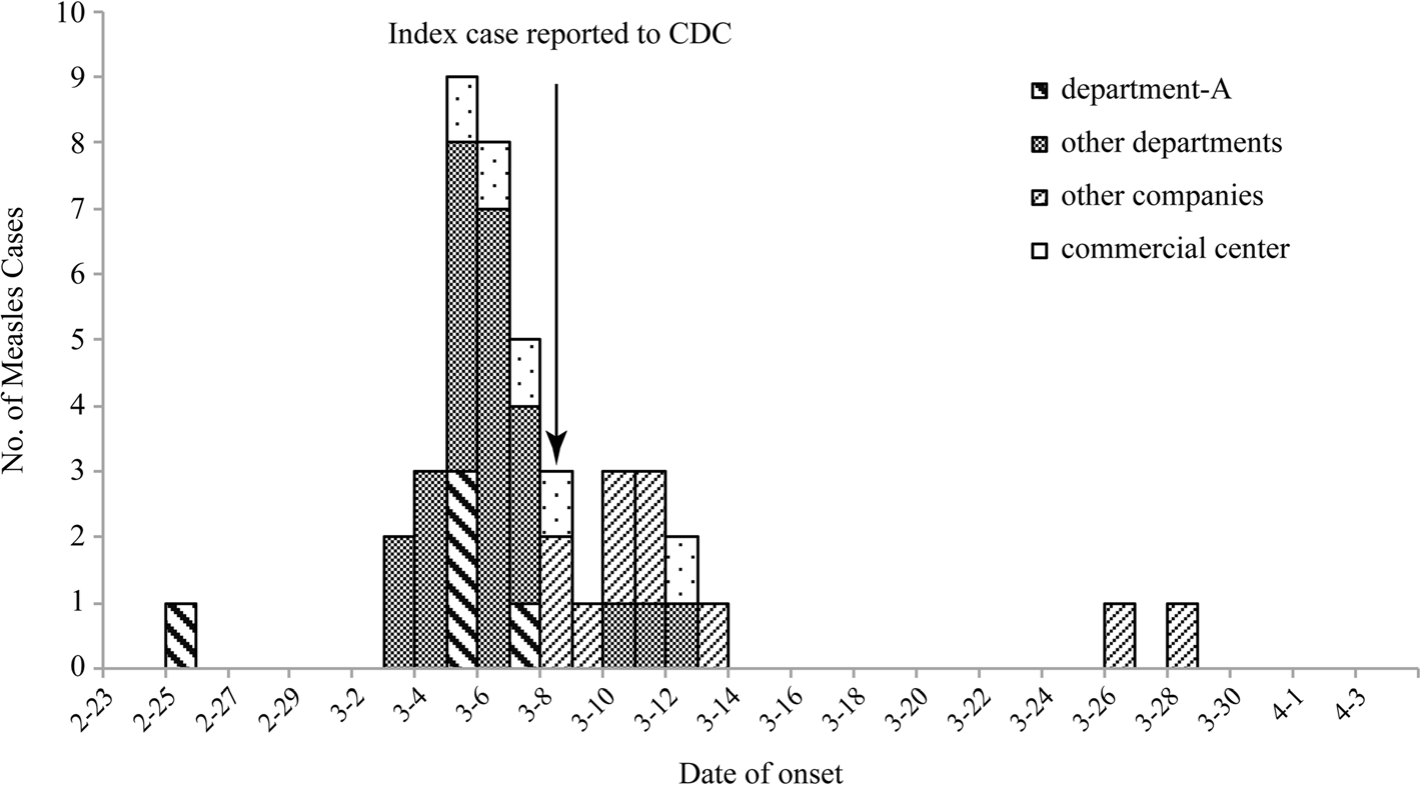
Epidemiologic Curve of 43 cases patients in the office building in Beijing, 2016

A total of 43 cases occurred between February 25 and March 28. The outbreak was over when there was no epidemiologically linked case within 21 days of the last case on March 28 (Fig. 1).

### Characteristics of measles cases

The median age of the 43 cases was 33 (range, 23–45) years. Among them, 26 (60.5%) were male and 30 (69.8%) were migrants (Table 1). Forty-three cases of measles resided in 9 of the 16 districts of Beijing, 20 of which lived in the districts where the outbreak took place, and 23 in the remaining 8 districts, with 1–6 cases per district.

**Table 1:** Characteristics of demography, symptoms and measles vaccination status among measles cases in Beijing, 2016

Variables	Value
Total(No.)	43
Age	
Range (years)	23–45
Median (years)	33
20~24 years (no. (%))	5 (11.6)
25~29 years (no. (%))	11 (25.6)
30~34 years (no. (%))	9 (20.9)
35~39 years (no. (%))	13 (30.2)
≥ 40 years (no. (%))	5 (11.6)
Sex (no. (%))	
Male	26 (60.5)
Female	17 (39.5)
Migrant	
Yes (no. (%))	30 (69.8)
No (no. (%))	13 (30.2)
Residence (no. (%))	
Chaoyang district^a^	20 (46.5)
Other districts of Beijing	23 (53.5)
Symptoms^b^(no. (%))	
Fever	42 (97.7)
Cough	19 (44.2)
Coryza	7 (16.3)
Conjunctivitis	12 (27.9)
Lymphadenectasis	7 (16.3)
Arthralgia	10 (23.3)
Complications^b^(no. (%))	3 (7.0)
Diarrhea	2 (4.7)
Pneumonia	1 (2.3)
Vaccination (no. (%))	
0 doses	17 (34.9)
1 dose	4 (18.6)
2 dose	1 (2.3)
Unknown	21 (44.2)

^a^It is the district where the outbreak took place

^b^Symptoms were self-reported. Complications were medically diagnosed, so that the complications of cases who didn’t go to hospital are unknown

Vaccination of all cases was obtained through patient memories without documentation. Five cases (11.6%) had previously received measles-containing vaccine (MCV) (Table 1). Four individuals who had received measles vaccine as part of the response went on to develop measles infection, two of whom had no prior doses and two had an unknown vaccination history.

Four (9.3%) had gone to a hospital and 16 (37.2%) had traveled to other cities 7–21 days before onset, suggesting that some cases may be infected elsewhere. Thirty-five cases (81.4%) had visited the doctor during the prodromal stage (no rash) and were infectious but could not be diagnosed. Twenty-three of the 43 cases had seen a doctor during the rash stage, only 19 (82.6%) had been reported measles to NNDRS. Therefore, it is speculated that about 56% of cases had not been reported by NNDRS.

### Laboratory results

All cases are laboratory diagnosed cases. The positive rates of measles-special-IgM and RT-PCR were 44.2% (19/43) and 81.4% (35/43) respectively. Seventeen cases of virus strains were isolated and identified as genotype H1.

### Attack rates

The crude attack rate of the measles outbreak was 1.20% (43/3596). The attack rate in the same company (or on the same floor) of the source case is 22.54 times that of other companies (other floors) in the office building (95%Confidence Interval (95%CI): 11.09, 45.82). The attack rate in the office building of the source case was 5.19 times higher than the associated commercial center (95%CI: 2.05, 13.17). However, there was no statistically significant difference of the attack rate between the department of the source case and other department in the company (2.11, (95%CI: 0.89, 5.01)) (Table 2).

**Table 2:** The comparisons of attack rates of different areas in the office building in Beijing, 2016

Variables	Effected population (No.)	Case Patients with measles (No.)	Attack rate (%)	Relative risk (95%CI)
All	3596	43	1.20	–
Office building	2136	38	1.78	5.19^c^ (2.05, 13.17)
Company A	236	28	11.86	22.54^a^ (11.09, 45.82)
Department A	22	5	22.73	2.11^b^ (0.89, 5.01)
Other departments	214	23	10.75	–
Other Companies	1900	10	0.53	–
Commercial center	1460	5	0.34	–

^a^The RR was company A compared with other companies

^b^The RR was same department compared with different departments

^c^The RR was office building compared with associated commercial center

### Outbreak control

Comprehensive measures were conducted in accordance with measles surveillance guidelines of the Beijing CDC [19], including cooperation among districts, Outbreak response immunization and active surveillance.

#### Active surveillance and case management

All measles cases are isolated at home or in hospital during their infectious period. In order to detect fever or rash patients, daily symptom monitoring was implemented in the office building and commercial center for 21 days (the maximum incubation period for measles) of the onset of the last case. At the same time, case monitoring was also carried out in the place of residence of each measles case for 21 days of the onset of the case. For those with fever, measures such as home isolation and clinical observation were taken, while the rash patients were managed according to suspected measles. Through daily symptom monitoring, 24 confirmed cases of measles had been found in the office building and the commercial center during the outbreak, but none in communities.

#### Outbreak response immunization

Those who were potentially in contact in the office building, the commercial center or the residence communities, aged 15–45 years and had not accepted measles-containing vaccine (MCV) or suffered from measles, received combined measles and rubella vaccines. From March 9 the outbreak response immunization (ORI) was implemented in Company A and exposed communities, then was extended to entire office building. From March 12 it was extended again to commercial center, where new case was found. From March 9 to 18, 6216 doses of MCV were administered, of which 3105 for the workplace and 3111 for residence communities. After March 18, no case was found in the accepted ORI population, but two cases occurred in the rejected ORI population in the office building.

#### Other

Response measures were conducted in the office building, including disinfection and increasing fresh air ventilation, to control spread. Group activities had been suspended until the outbreak was declared over.

## Discussion

Since measles elimination efforts began in 2006, significant reduction of measles incidence has been achieved in Beijing; down 76%, from 244.5 per million in 2006 to 57.9 per million in 2016 [4, 5]. However, the characteristics of measles epidemics in Beijing have conspicuously changed in recent years with outbreak settings shifting, for instance, from factories and large-scale markets to office buildings, and the subject profiles from migrant laborers to white-collar workers [3, 12, 13, 20]. The outbreak of the office buildings reported here was the largest in Beijing, which had lasted 34 days. Furthermore, the nucleotide sequence was analyzed and showed that genotype H1 viruses detected in 17 cases were the dominant virus strain and had been circulating continuously in China [18, 21].

There were a few notable features of this outbreak. Firstly, the space of the building, with a central air conditioning system, was relatively closed and crowded, facilitating respiratory diseases and epidemics [3, 20]. Secondly, the white-collar workers working in office buildings are mostly 25–45 years old. Due to immunization gaps or immune failure, they became the high risk population of measles in Beijing in recent years [3, 22–25]. The accumulation of susceptible individuals can easily cause large outbreaks. Finally, most of office buildings are located in the city center, while most staffs live in surrounding areas of the city. If these cases were diagnosed too late, there would be a transmission risk to communities.

The incident risk in different areas of the outbreak has been evaluated. The attack rate in the same company (or on the same floor) of the source case is 22.54 times of other companies (other floors) in the office building (95%CI: 11.09, 45.82); which may be mainly related to exposure opportunities. Company A occupies the entire 11th floor. People in the same company or on the same floor of the source case may have more exposure opportunities during working hours, especially in the same department. The exposures for people on other floors are more likely to occur during sharing of elevator with the source case, and were considered close contacts but occasional. Although the risk of illness in the associated commercial center is lower than in the office building, it suggests that dining and shopping in the commercial center may cause spread.

The outbreak shows that the following measures should be taken when an outbreak occurs in office buildings. Firstly, ORI should be carried out as soon as possible, and the history of outside activities such as meals and shopping during the infectious period should be investigated in detail. ORI should cover populations with potential exposure, even if it does not protect those who have already exposed, but can protect against subsequent exposures. Secondly, active surveillance must be implemented. Fifty-six percent of all cases in the outbreak were not reported by hospital. If these unreported cases had not been detected as early as possible, outbreaks could not have been effectively controlled. Thirdly, during the medical observation period, cases of fever or rash symptoms should be isolated immediately, which can effectively reduce the transmission risk of potential measles patients [20].

The outbreak highlighted ongoing challenges to achieving elimination, including sensitivity of the surveillance system and immunity gaps among adults. Delayed recognition of measles cases could have meant that the optimal time for controlling spread was missed, leading to an even larger outbreak [26–29]. The source case had been sick on February 25, but had not been found until March 8, when the index case was reported by NNDRS and the Beijing Infectious Symptoms Surveillance System, alerting us to the measles outbreak and enabling implementation of response measures. Earlier recognition of the correct diagnosis and source of infection might have led to better measles control and cost savings [20, 30, 31]. To achieve measles elimination, measures are needed to maintain high-quality surveillance for rapid case detection [32, 33], so clinicians should keep measles in their differential diagnosis of febrile rash illness for rapid case detection. Adults had become the high risk population of measles in Beijing and similar areas [3, 5, 18, 22–26], suggesting that there are immunity gaps to measles among adults. During outbreaks, ORI can effectively close immunity gaps among children [34, 35], as well as among adults in China [36, 37]. In addition, there is no guideline of adult vaccinations for measles in Beijing. The 2013 US guidelines can be referred to recommending two doses for adults at high risk for exposure and transmission and 1 dose for other adults aged ≥18 years [38].

There are also limitations in this outbreak investigation. No exposure evaluation was conducted, and the exposure risk was indirectly evaluated through the attack rates. It is recommended that future outbreaks should investigate all possible exposures and determine exposures through monitoring systems as necessary [39]. In addition, most adult vaccinations had not been documented in China. When an outbreak occurs, the coverage rate and effectiveness of the vaccine among adults cannot be evaluated. The nucleotide sequencing of the virus was undertaken in 17 cases, therefore, the cases in the outbreak may have originated from other infectious sources, such as within the commercial center.

## Conclusions

Office buildings in densely populated metropolitan are prone to appear large-scale outbreaks, which should be paid attention to. The highest attack rate in office buildings is in the company or floor where the source case worked during the infectious period. The elevator may be an important exposure point, causing the spread among floors [26]. Early outbreak response immunization and active surveillance are effective for controlling outbreaks. In order to reduce adult morbidity, it is recommended that vaccination guideline for adult be developed to improve the vaccination coverage of MCV.

## Data Availability

The datasets used and/or analyzed during the current study are available from the corresponding author on reasonable request.

## Abbreviations

CI: Confidence Interval
ELISA: enzyme linked immunosorbent assay
IgG: Immunoglobulin G
MCV: measles-containing vaccine
NNDRS: National Notifiable Disease Reporting System
ORI: Outbreak response immunization
RR: Relative Risk
RT-PCR: real-time reverse transcription polymerase chain reaction
SIAs: Supplementary immunization activities
